# The GATOR1 Complex Regulates Metabolic Homeostasis and the Response to Nutrient Stress in *Drosophila melanogaster*

**DOI:** 10.1534/g3.116.035337

**Published:** 2016-09-26

**Authors:** Youheng Wei, Brad Reveal, Weili Cai, Mary A. Lilly

**Affiliations:** Cell Biology and Neurobiology Branch, National Institute of Child Health and Human Development, National Institutes of Health, Bethesda, Maryland 20892

**Keywords:** TORC1, Nprl2, Nprl3, Iml1, metabolism

## Abstract

TORC1 regulates metabolism and growth in response to a large array of upstream inputs. The evolutionarily conserved trimeric GATOR1 complex inhibits TORC1 activity in response to amino acid limitation. In humans, the GATOR1 complex has been implicated in a wide array of pathologies including cancer and hereditary forms of epilepsy. However, the precise role of GATOR1 in animal physiology remains largely undefined. Here, we characterize null mutants of the GATOR1 components *nprl2*, *nprl3*, and *iml1* in *Drosophila melanogaster*. We demonstrate that all three mutants have inappropriately high baseline levels of TORC1 activity and decreased adult viability. Consistent with increased TORC1 activity, GATOR1 mutants exhibit a cell autonomous increase in cell growth. Notably, escaper *nprl2* and *nprl3* mutant adults have a profound locomotion defect. In line with a nonautonomous role in the regulation of systemic metabolism, expressing the Nprl3 protein in the fat body, a nutrient storage organ, and hemocytes but not muscles and neurons rescues the motility of *nprl3* mutants. Finally, we show that *nprl2* and *nprl3* mutants fail to activate autophagy in response to amino acid limitation and are extremely sensitive to both amino acid and complete starvation. Thus, in *Drosophila*, in addition to maintaining baseline levels of TORC1 activity, the GATOR1 complex has retained a critical role in the response to nutrient stress. In summary, the TORC1 inhibitor GATOR1 contributes to multiple aspects of the development and physiology of *Drosophila*.

The Target of Rapamycin Complex 1 (TORC1) regulates nutrient sensing and cell metabolism from yeast to humans (Loewith and Hall 2011; Laplante and Sabatini 2012). At the heart of the TORC1 complex is the serine/threonine kinase Tor (Schmelzle and Hall 2000). In the presence of sufficient nutrients and growth signals, TORC1 is active and stimulates protein synthesis and cell growth through the phosphorylation of downstream effectors such as S6K and 4E-BP, while simultaneously inhibiting catabolic metabolism and autophagy (Hay and Sonenberg 2004; Wullschleger *et al.* 2006). Conversely, when nutrient or growth factors are limiting, TORC1 is inactivate resulting in the inhibition of cell growth and the promotion of catabolic metabolism (He and Klionsky 2009; Jung *et al.* 2010). Thus, by modulating the activity of TORC1, cells can rapidly adjust their metabolic state in response to both extracellular and intracellular stimuli.

Mutations in upstream signaling pathways that regulate TORC1 result in a wide array of human pathologies. Many of these pathologies, such as the development of benign tumors and a predisposition to cancers, are associated with increased TORC1 activity and cell growth (Laplante and Sabatini 2012). TORC1 activity also contributes to numerous age-related diseases including cancer, diabetes, and neurodegenerative disorders such as Parkinson’s (Laplante and Sabatini 2012; Johnson *et al.* 2013). Reducing TORC1 activity through genetic, pharmacological, or nutritional intervention extends lifespan across multiple model organisms including yeast, *Caenorhabditis elegans*, *Drosophila*, and mice, while increasing TORC1 activity results in decreased lifespan (Evans *et al.* 2011; Johnson *et al.* 2013; Fontana and Partridge 2015). Recent evidence indicates that mutations that inactivate several upstream inhibitors of TORC1 result in the development of focal epilepsies via an unknown mechanism (Dibbens *et al.* 2013; Tee *et al.* 2016). Thus, the precise regulation of TORC1 activity is critical to multiple aspects of human health.

Two GTPases, Rheb and the Rags, play a key role in the regulation of TORC1 activity. The small GTPase Rheb activates TORC1 on the surface of lysosomes (Yang *et al.* 2006; Sancak *et al.* 2008). The Rag GTPase consists of four proteins RagA, RagB, RagC, and RagD, that function as heterodimers (Kim *et al.* 2008; Sancak *et al.* 2008). While amino acids are sufficient, RagA/B binds GTP and RagC/D binds GDP. In this active configuration, the Rags function as GTPases that promote the recruitment of TORC1 to lysosomes where it encounters its activator Rheb. Thus, an important step in the activation of TORC1 is the Rag GTPase-dependent recruitment of the TORC1 complex to lysosomes.

Recently the GAP activity toward Rags (GATOR) complex, which is named the Seh1 associated (SEA) complex in yeast, was shown to regulate TORC1 activity through the Rag GTPases (Dokudovskaya *et al.* 2011; Wu and Tu 2011; Bar-Peled *et al.* 2013; Panchaud *et al.* 2013a). Iml1/DEPDC5, Nprl2, and Nprl3 comprise GATOR1, which functions as a GTPase activating protein (GAP) for RagA/B, and thus acts as an inhibitor of TORC1 activity. The GATOR1 complex is called the SEA Complex Inhibits TORC1 (SEACIT) in yeast (Dokudovskaya *et al.* 2011; Panchaud *et al.* 2013b). Deletion mutants of the SEACIT/GATOR1 components *npr2*, *npr3*, and *iml1* have a reduced ability to grow on a poor nitrogen source or restricted methionine, but do not have proliferation defects or increased TORC1 activity under conditions of amino acid sufficiency (Neklesa and Davis 2009; Ma *et al.* 2013). In contrast, in mammalian and *Drosophila* tissue culture cells, depleting GATOR1 components results in a dramatic increase in TORC1 activity under standard culture conditions (Bar-Peled *et al.* 2013; Wei and Lilly 2014). Notably, recent reports have shown that GATOR1 knockouts of iml1/Depdc5 (rat), *nprl2* (mouse), and *nprl3* (mouse) result in late embryonic lethality, with embryos exhibiting developmental defects in the heart, liver, and brain (Kowalczyk *et al.* 2012; Dutchak *et al.* 2015; Marsan *et al.* 2016). Additionally, in *Drosophila*, the GATOR1 complex regulates entry into the meiotic cycle during oogenesis (Wei *et al.* 2014). Taken together, these data strongly suggest that, in metazoans, the GATOR1 complex has evolved roles beyond regulating an adaptive response to amino acid starvation.

Here, we demonstrate that the GATOR1 complex has both cell autonomous and nonautonomous effects on growth and metabolism in the model organism *Drosophila melanogaster*. We demonstrate that the GATOR1 complex mutants have increased baseline levels of TORC1 activity and decreased viability. Moreover, we show that GATOR1 mutants exhibit multiple phenotypes consistent with increased TORC1 activity including increased cell growth, reduced tolerance to starvation, and the inability to activate autophagy under nutrient-limiting conditions. Intriguingly, GATOR1 mutants exhibit a serious locomotion deficit that can be rescued by altering systemic metabolism. Taken together, our data demonstrate that *Drosophila* will provide an excellent model to study how GATOR1 influences both development and disease in multicellular animals.

## Materials and Methods

### Fly stocks

The stocks Df(1)*BSC582*, *w^1118^/Binsinscy* (BDSC#25416), *w^1118^*; *Df(3L)ED4515*, *P{3′.RS5+3.3′}ED4515/TM6C*, *cu^1^ Sb^1^* (BDSC#9071,), *w1118*; *Df(3L)ED4238*, *P{3′.RS5+3.3′}ED4238/TM6C*, *cu^1^ Sb^1^* (BDSC#8052), w*; *P{GAL4-elav.L}3* (BDSC#8760), *w^1118^*; *P{Cg-GAL4.A}2* (BDSC#7011), *P{Ubi-mRFP.nls}1*, w*, *P{hsFLP}12P{neoFRT}19A* (BDSC#31418), *P{neoFRT}19A ry^506^* (BDSC#1709), w*; *P{neoFRT}80B ry^506^* (BDSC#1988), and w*; *P{w[+mW.hs]=GAL4-da.G32}2*; *MKRS/TM6B*, *Tb[1]* (BDSC#55851) were obtained from Bloomington Stock Center. *y,w*; *Tor ^A948V^/CyO* (Zhang *et al.* 2006) was kindly provided by Thomas P. Neufeld (University of Minnesota). All fly stocks were maintained on JAZZ-mix *Drosophila* food (Fisher Scientific, Waltham, MA) at 25°. For complete starvation, flies were cultured on media consisting of phosphate-buffered saline (PBS) and 0.8% agar. For amino acid starvation, flies were cultured on media consisting of PBS, 20% sucrose, and 0.8% agar.

### Generation of nprl2 and iml1 deletion mutants

Guide RNAs (gRNA) that target *nprl2* and *iml1* were designed using the online CRISPR design tool (http://crispr.mit.edu/). To make the deletion mutants, two sets of gRNAs that target each gene were cloned into pBFv-U6.2B as previously described (Kondo and Ueda 2013). The two gRNA were separately expressed from their own U6 promoters in one plasmid. The pBFv-U6.2B-nprl2 and pBFv-U6.2B-iml1 plasmids were separately injected into *w^[1118]^*; *vas-Cas9* (BDSC#51324) and *y^[1]^*, *vas-Cas9*, *w^[1118]^* (BDSC#52669) embryos (Rainbow Transgenic Flies, Inc.). Eclosed flies were individually crossed with *y*, *w* and screened by PCR using genomic DNA. The offspring of positive screens were balanced to generate *nprl2* and *iml1* mutant stocks. Primers are listed in Supplemental Material, Table S1.

### Generation of nprl2, nprl3, and iml1 transgenic lines

The pENTR-Nprl2 and pENTR-Nprl3 plasmids were recombined into pPFHW vectors (DGRC) to generate UASp-3×FLAG-3×HA-Nprl2 and UASp-3×FLAG-3×HA-Nprl3 plasmids using the Gateway LR Clonase II Enzyme (Invitrogen) (Wei and Lilly 2014). The fragments that contained 837 bp upstream of the *iml1* transcription start, the *iml1* 5′UTR, the *iml1* coding region, and the EGFP coding region were amplified by PCR and cloned into the attB-P[acman]-Cm^R^-BW vector. The UASp-3×FLAG-3×HA-Nprl2, UASp-3×FLAG-3×HA-Nprl3, and attB-P[acman]-Cm^R^-BW-Iml1 plasmids were used to generate transgenic lines (Best Gene Inc.).

### Lethal phase analysis

Homozygous *nprl2^1^*, *nprl3^1^/Df*, and *iml1^1^/Df* first-star larvae were distinguished from *nprl2^1^/FM7-GFP*, *nprl3^1^/TM3-GFP*, and *iml1^1^/TM3-GFP* based on the absence of GFP. First-star larvae were collected and cultured in new vials at a density of 50 larvae per vial. The number of larvae that developed to the pupae and adult stage was determined for each genotype.

### Geotaxis motility assay

Geotaxis motility assay was performed essentially as described by Ganetzky and Flanagan (1978). Briefly, 10–15 flies were placed in a 10 cm vial. The flies were tapped to the bottom of the vial and given 15 sec to climb from the bottom to top. The number of flies arriving at the top were counted and divided by the number of total flies in the vial. Each analysis was repeated five times to obtain an average value.

### Clonal analysis

*HS-FLP*, *UAS-RFP FRT19A/nprl2^1^ FRT19A*, *HS-FLP*; *Ubi-GFP FRT80B/nprl3^1^ FRT80B* and *HS-FLP*; *Ubi-GFP FRT80B/iml1^1^ FRT80B* flies were used to generate mutant clones in the germline and somatic cells of the ovary as previously reported (Wei *et al.* 2014). Briefly, female flies were heat-shocked for 2 hr at 37° each day for 3 d. Subsequently, flies were cultured in standard media for an additional 2 d before dissection. Homozygous mutant clones were identified by the absence of GFP or RFP.

### Western blots

*Drosophila* third-star larvae were homogenized in RIPA buffer containing complete protease inhibitors and phosphatase inhibitors (Roche). Western blots were performed as described previously (Wei *et al.* 2014). Antibodies were used at the following concentrations: rabbit anti-P-S6K at 1:500 (Cell Signaling) and guinea pig anti-S6K at 1:10,000 (Hahn *et al.* 2010). Band intensity was quantified using Image J (NIH).

### Immunofluorescence and confocal microscopy

Immunofluorescence staining and microscopy was performed as described previously (Hong *et al.* 2003; Iida and Lilly 2004; Senger *et al.* 2011), using a mouse anti-1B1 (1:30, Developmental Studies Hybridoma Bank) antibody. Anti-mouse Alexa Fluor secondary antibodies (Invitrogen) were used at a dilution of 1:1000. Nuclei were visualized by staining the DNA with 4′,6-diamidino-2-phenylindole (DAPI) (Invitrogen). Images were acquired using a Leica TCS SP5 confocal microscope. Follicle cell size was quantified using Image J (NIH) based on 1B1 staining.

### Data availability

All strains are available upon request. The authors state that all data necessary for confirming the conclusions presented in the article are represented fully within the article.

## Results and Discussion

### The GATOR1 components Nprl2, Nprl3, and Iml1 are required for viability in Drosophila

**Figure 1:**
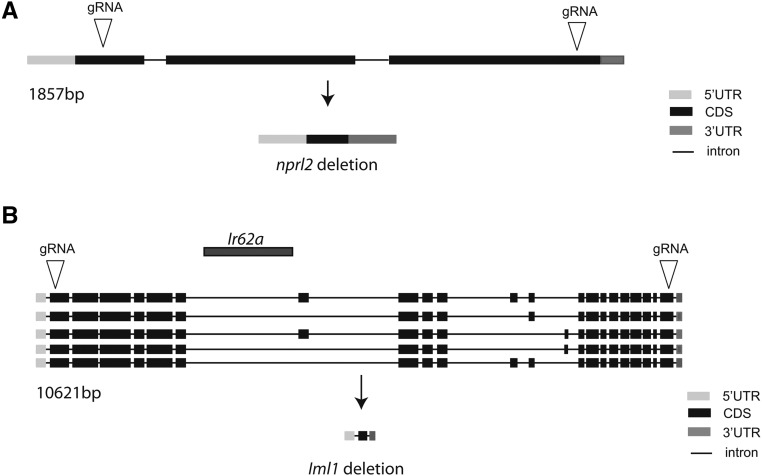
Schematic representation of GATOR1 mutants generated using CRISPR/Cas9 genome editing. To generate the *nprl2* and *iml1* deletion mutants, two gRNAs that target the 5′ and 3′ of each gene were used. (A) *nprl2* encodes a transcript of 1707 bp and a protein of 412 amino acids (FBgn0030800). The *nprl2^1^* deletion contains 30 amino acids from the N-terminus and four frame-shifted amino acids from the C-terminus. (B) *iml1* encodes five transcripts (FBgn0035227). These transcripts encode proteins of 1471, 1472, 1503, 1511, and 1544 amino acids, respectively. The *iml1^1^* deletion contains 17 amino acids from the N-terminus and 53 amino acids from the C-terminus of all *iml1* transcripts. Notably, the intron of *iml1* also encodes a transcript Ir62a, which is also deleted. CDS, coding sequences; CRISPR, clustered regularly interspaced short palindromic repeats; gRNA, guide RNA; UTR, untranslated region.

To evaluate the physiological roles of GATOR1 *in vivo*, we generated *nprl2* and *iml1* deletion alleles using CRISPR/Cas9 (Kondo and Ueda 2013). We had previously generated *nprl3* deletion mutants but had not fully evaluated the mutant phenotype (Cai *et al.* 2016). In each gene, > 90% of the coding region was deleted (Figure 1). For each gene, multiple alleles were generated that displayed similar phenotypes. For studies here, we used the *nprl2^1^*, *nprl3^1^*, and *iml1^1^ alleles*. In order to define the requirement for individual GATOR1 complex components, we examined the viability of individual *GATOR1* deletion mutants. Notably, both *nprl2^1^* and *nprl3^1^* homozygous adults eclosed at significantly reduced rates (26 and 9% of Mendelian expectations, respectively), while no *iml1^1^* homozygous adults eclosed (Table 1). Thus, deletions of *nprl2* and *nprl3* are semilethal while deletions of *iml1* are lethal. To rule out the possibility that the lethality of *nprl2^1^*, *nprl3^1^*, and *iml1^1^* were the result of the genetic background, we examined each deletion mutant *in trans* to the appropriate genomic deficiency (Table 1). We determined that the transheterozygotes *nprl2^1^/Df* and *iml1^1^/Df* eclosed at rates similar to the respective *nrpl2^1^* and *iml1^1^* homozygotes. These data are consistent with the *nprl2* and *iml1* mutants being null alleles. However, *nrpl3^1^/Df* transheterozygotes eclosed at a significantly higher rate than *nprl3^1^* homozygous mutants (24 *vs.* 9%, respectively). These data suggest that at least some of the lethality associated with the *nprl3* deletion may be due to genetic background.

**Table 1:** Lethality of GATOR1 mutants

Cross	Genotype (Number of Adult Flies)		% of Expected Ratio
*nprl2^1^* (Male) × *nprl2^1^/FM7* (Female)	*nprl2^1^/FM7* (Female)	*nprl2^1^* (Female)	26.3*^a^*
	388	102	
*nprl2^1^* (Male) × *Df/FM7* (Female)	*nprl2^1^/FM7* (Female)	*nprl2^1^/Df* (Female)	28.1*^a^*
	203	57	
*nprl3^1^/TM3* × *nprl3^1^/TM3*	*nprl3^1^/TM3*	*nprl3^1^*	9.4*^b^*
	641	30	
*nprl3^1^/TM3* × *Df/TM3*	*nprl3^1^/TM3* and *Df/TM3*	*nprl3^1^/Df*	24.1*^b^*
	705	85	
*nprl2^1^/FM7*; *nprl3^1^/TM3* (Male) × *Df/TM3* (Female)	*nprl3^1^/TM3* (Female) and *Df/TM3* (Female)	*nprl2^1^*; *nprl3^1^/Df* (Male)	29.4*^c^*
	571	42	
*iml1^1^/TM3* × *iml1^1^/TM3*	*iml1^1^/TM3*	*iml1^1^*	0*^b^*
	1200	0	
*iml1^1^/TM3* × *Df/TM3*	*iml1^1^/TM3* and *Df/TM3*	*iml1^1^/Df*	0.5*^b^*
	387	1	

a In these crosses, the FM7 (first chromosome balancer) was identified by the *Bar* eye. The expected Medelian ratio of non-*Bar* eyes to *Bar* eye female flies was 1:1.

b In these crosses, the TM3 (third chromosome balancer) was identified by the *Sb* marker. The expected Medelian ratio of non-*Sb* to *Sb* flies was 1:2, since the TM3/TM3 is embryonic lethal.

c In these crosses, the FM7 and TM3 was identified by the *Bar* and *Sb* markers. The expected Medelian ratio of male non-*Sb* and non-*Bar* to female *Sb* (both *Bar* and non-*Bar*) flies was 1:4, since the TM3/TM3 is embryonic lethal.

To demonstrate formally that the lethality of the mutants was due to the deletion of the GATOR1 genes, we overexpressed HA-tagged Nprl2 and Nprl3 using the Ubi-GAL4 driver and GFP-tagged Iml1 using the native *iml1* promoter in the *nprl2*, *nprl3*, and *iml1* mutant backgrounds, respectively. As shown in Table 2, the expression of each GATOR1 protein fully rescued the lethality associated with the respective GATOR1 mutant. Taken together, our data support the conclusion that null alleles of *nrpl2* and *nprl3* are semilethal while null mutants of *iml1* are nearly fully lethal. Thus, in *Drosophila*, all three components of the GATOR1 complex are required for full viability.

**Table 2:** Rescue of GATOR1 mutants’ lethality

Cross	Genotype (Number of Adult Flies)		% of Expected Ratio
*nprl2^1^*; *UAS-Nprl2* (male) × *nprl2^1^/FM7*; *Ubi-GAL4* (Female)	*nprl2^1^/FM7;Ubi > Nprl2* (Female)	*nprl2^1^*; *Ubi > Nprl2* (Female)	111.6*^a^*
	155	173	
*Ubi-GAL4*; *nprl3^1^/TM3* × *UAS-Nprl3*; *nprl3^1^/TM3*	*Ubi > Nprl3*; *nprl3^1^/TM3*	*Ubi > Nprl3*; *nprl3^1^*	109.3*^b^*
	302	165	
*iml1-Iml1/SM6*; *iml1^1^/TM3* × *iml1^1^/TM3*	*iml1-Iml1*; *iml1^1^/TM3*	*iml1-Iml1*; *iml1^1^*	102.0*^c^*
	394	201	
*Ubi-GAL4*; *nprl3^1^/TM3* × *UAS-Nprl3*; *Df/TM3*	*Ubi > Nprl3*; *nprl3^1^/TM3* and *Ubi > Nprl3*; *Df/TM3*	*Ubi > Nprl3*; *nprl3^1^/Df*	113.6*^b^*
	405	230	
*iml1-Iml1/SM6*; *iml1^1^/TM3* × *Df/TM3*	*iml1-Iml1*; *iml1^1^/TM3* and *Iml1*; *Df/TM3*	*iml1-Iml1*; *iml1^1^/Df*	106.7*^c^*
	283	151	
*nprl2^1^*; *Tor^AV^/SM6* (Male) × *nprl2^1^/FM7* (Female)	*nprl2^1^/FM7;Tor^AV^/+* (Female)	*nprl2^1^*; *Tor^AV^/+* (Female)	89.7*^d^*
	290	260	
*Tor^AV^/SM6*; *nprl3^1^/TM3* × *Tor^AV^/SM6*; *Df/TM3*	*Tor^AV^/+*; *nprl3^1^/TM3* and *Tor^AV^/+*; *Df/TM3*	*Tor^AV^/+*; *nprl3^1^/Df*	90.7*^c^*
	344	156	
*CG-GAL4*; *nprl3^1^/TM3* × *UAS-Nprl3*; *Df/TM3*	*CG > Nprl3*; *nprl3^1^/TM3* and *CG > Nprl3*; *Def/TM3*	*CG > Nprl3*; *nprl3^1^/Df*	125.3*^b^*
	241	151	

a In these crosses, the FM7 (first chromosome balancer) was identified by the *Bar* eye. The expected Medelian ratio of non-*Bar* eyes to *Bar* eye female flies was 1:1.

b In these crosses, the TM3 (third chromosome balancer) was identified by the *Sb* marker. The expected Medelian ratio of non-*Sb* to *Sb* flies was 1:2, since the TM3/TM3 is embryonic lethal.

c In these crosses, the SM6 (second chromosome balancer) and TM3 was identified by the *Cy* and *Sb* markers. The expected Medelian ratio of non-*Cy* and non-*Sb* to non-*Cy* and *Sb* flies was 1:2, since the TM3/TM3 is embryonic lethal.

d In these crosses, the FM7 and SM6 was identified by the *Bar* and *Cy* markers. The expected Medelian ratio of non-*Cy* and non-*Bar* to non-*Cy* and *Bar* female flies was 1:1.

There are at least two possible models to explain why *iml1* has a stronger phenotype than either *nprl2* or *nprl3*. First, the Iml1 protein may retain some GAP activity toward Rags or another critical activity in the *nprl2* and *nprl3* mutant background. In this model, Iml1 functions, at least in part, independently of Nprl2 and Nprl3. Consistent with the first model, the overexpression of Iml1 in yeast bypasses the requirement for the Npr2 and Npr3 proteins, suggesting that the enzymatic component of the GATOR1 complex resides within the Iml1 protein (Panchaud *et al.* 2013a). A second possible model is that Nprl2 and Nprl3, which share a similar domain structure (Dokudovskaya *et al.* 2011), are functionally redundant. Both Nprl2 and Nprl3 contain a C-terminal Longin domain, which is often found in proteins that regulate membrane trafficking (Levine *et al.* 2013), followed by two or three N-terminal helix-turn-helix domains (Zhang *et al.* 2012; Levine *et al.* 2013). To test if *nprl2* and *nprl3* are redundant, we generated *nprl2* and *nprl3* double mutants. We reasoned that if *nprl2* and *nprl3* are redundant, then the double mutants should have a stronger phenotype, as measured by lethality rates, than either single mutant. However, we found that *nprl2*, *nprl3* double-mutants eclosed at approximately the same rate as *nprl2* and *nprl3* single mutants (Table 1). Thus, although structurally similar, the Nprl2 and Nprl3 proteins are not functionally redundant. Moreover, the stronger phenotype observed in the *iml1^1^* mutants is consistent with the Iml1 protein of *Drosophila* retaining critical activities independent of the GATOR1 components Nprl2 and Nprl3.

In order to determine the specific developmental stage at which the GATOR1 mutants die, newly hatched first instar larvae were cultured and counted at both pupal and adult stages. As shown in Figure S1, *nprl2*, *nprl3*, and *iml1* mutant larvae advance to the pupae stage at rates similar to those observed for wild-type larvae. However, only 37% of *nprl2^1^*, 36% of *nprl3^1^/Df*, and 2% of *iml1^1^/Df* mutant larvae ultimately eclosed. In contrast, 82% wild-type larvae eclosed. Thus, the activity of the GATOR1 complex is required to transit from the pupal to the adult stage of development. We noticed that the dead nprl2^1^, nprl3^1^/Df, and iml1^1^/Df pupae had finished metamorphosis and died beyond the pharate adult stage P13, as determined by the presence of black wings (Bainbridge and Bownes 1981). Thus, the GATOR1 complex is required for animals to transit the last stage of pupal development and eclose as adults.

### GATOR1 maintains baseline levels of TORC1 activity in vivo

**Figure 2:**
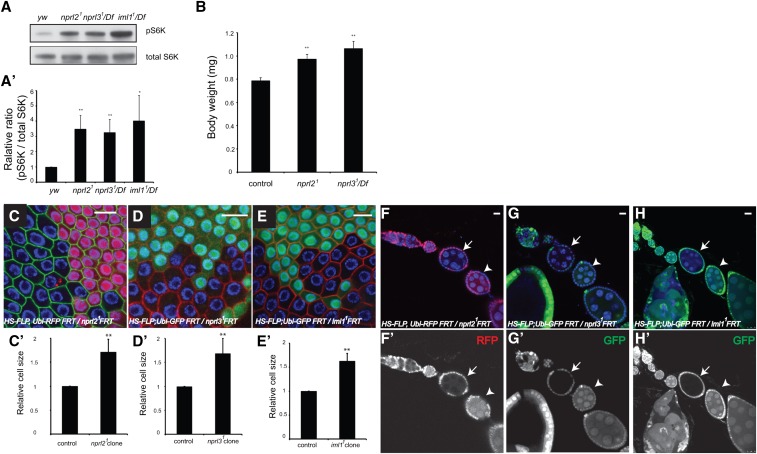
GATOR1 is required to maintain baseline levels of TORC1 activity in *Drosophila*. (A) Third star larvae were lysed in RIPA buffer. The protein levels of phospho-T398-S6K and total S6K were determined by western blot. (A’) The ratio of pS6K/S6K in the *y*, *w* lysis was set as 1. Error bars indicate SD of three independent experiments. * *P* < 0.05, ** *P* < 0.01. (B) The body weight of male flies. Error bars indicate SD of five independent experiments. ** *P* < 0.01. (C–E) Ovaries were dissected and stained with DAPI and anti-1B1 antibody. The *nprl2^1^*, *nprl3^1^*, and *iml1 ^1^* homozygous follicles cell clones were identified by the absence of RFP or GFP. The 1B1 antibody stains cell membranes and was used to mark the outline of the cells (C’–E’). The area of homozygous cells and adjacent wild-type cells were quantified using Image J. The mean value of the wild-type cell size was set as 1 for each image. For each genotype, eight images were quantified. Error bars indicate SD. ** *P* < 0.01. (F–H) Ovaries were dissected and stained with DAPI. The *nprl2^1^*, *nprl3^1^*, and *iml1 ^1^* homozygous egg chamber clones were identified by the absence of RFP and GFP. Homozygous mutant cells are indicated by an arrow, while older wild-type cells are indicated by an arrowhead. Note that younger homozygous mutant cells are larger than older wild-type cells. (F’–H’) In order to clearly show the clonal boundary, RFP and GFP channels are shown separately. DAPI, 4′,6-diamidino-2-phenylindole; GFP, green fluorescent protein; RFP, red fluorescent protein; RIPA, radioimmunoprecipitation assay.

In yeast, GATOR1 downregulates TORC1 activity in response to amino acid restriction but is not required to maintain baseline levels of TORC1 activity under nutrient rich conditions (Neklesa and Davis 2009). In contrast, in mice and rats, components of the GATOR1 complex are required for viability, strongly suggesting that, in metazoans, the GATOR1 complex has evolved roles beyond the response to nutrient stress (Table 1) (Kowalczyk *et al.* 2012; Dutchak *et al.* 2015; Marsan *et al.* 2016). In order to determine if the GATOR1 complex is required to maintain baseline levels of TORC1 activity *in vivo* in *Drosophila*, we measured phosphorylation of S6 Kinase (S6K), a direct readout for TORC1 activity, in well-fed whole animal lysates by western blot using an anti-phosopho-Thr398 S6K antibody (Hara *et al.* 1998). From these experiments, we found that the levels of phospho-T398-S6K were significantly increased in lysates from *nprl2*, *nprl3*, and *iml1* mutants (Figure 2A). Thus, unlike what is observed in single-celled eukaryotes, in *Drosophila* the GATOR1 complex is required to maintain baseline levels of TORC1 activity *in vivo* under nutrient-replete conditions. Similarly, rat and mouse cells cultured from *nprl2*, *nprl3*, or *iml1/Depdc5* mutants have increased TORC1 activity relative to controls (Kowalczyk *et al.* 2012; Dutchak *et al.* 2015; Marsan *et al.* 2016). Thus, in both *Drosophila* and mammals, the GATOR1 complex is required to maintain baseline levels of TORC1 activity independent of nutrient status.

We reasoned that the lethality observed in GATOR1 mutants is due to the hyperactivation of TORC1. To test our hypothesis we used the mutation *Tor^A948V^*, which interferes with wild-type Tor function (Zhang *et al.* 2006). Notably, placing one copy of the *Tor^A948V^* allele in the *nprl2* or *nprl3* mutant backgrounds resulted in eclosion rates that were three-fold higher than *nprl2* and *nprl3* mutants that contained two functional copies of the Tor gene (Table 2). These data confirm that the lethality associated with *nprl2* and *nprl3* mutations is due to the hyperactivation of TORC1.

### GATOR1 inhibits cell growth in multiple cell types

TORC1 is a potent activator of cell growth (Laplante and Sabatini 2012; Tee 2014). Consistent with the overall increase in TORC1 activity observed in whole animals, *nprl2* and *nprl3* escaper adults exhibit a small increase in body weight relative to wild-type controls (Figure 2B). In order to determine if the increased growth observed in GATOR1 mutants is at least in part due to a cell autonomous requirement for the GATOR1 complex, we used the FLP/FRT system to generate homozygous *nprl2*, *nprl3*, or *iml1* mutant clones in multiple tissues (Theodosiou and Xu 1998). In the *Drosophila* ovary, developing egg chambers contain a 16-cell germline cyst that is comprised of 15 large polyploid nurse cells and a single oocyte (de Cuevas *et al.* 1997). During oogenesis, the polyploid nurse cells grow via endoreplication and are responsible for providing the oocyte with nutrients. The growing germline cyst is surrounded by a layer of somatically-derived follicle cells that also increase in size through the process of endoreplication (Lilly and Duronio 2005). Notably, TORC1 activity is an important factor driving endoreplication and growth in polyploid tissues (Saucedo *et al.* 2003). We found that homozygous mutant *nprl2^1^*, *nprl3^1^*, and *iml1^1^* follicle cells were larger than adjacent heterozygous cells (Figure 2, C–E). Similarly, egg chambers that contained homozygous mutant germline clones (arrow) of *nprl2*, *nprl3*, or *iml1* were significantly increased in size relative to older egg chambers (arrowhead) that contained a wild-type heterozygous germline (Figure 2, F–H). Taken together, these data are consistent with the GATOR1 complex having cell autonomous effects on TORC1 activity and cell growth *in vivo*.

### Nprl2 and Nprl3 promote starvation resistance, starvation-induced autophagy, and triglyceride storage

**Figure 3:**
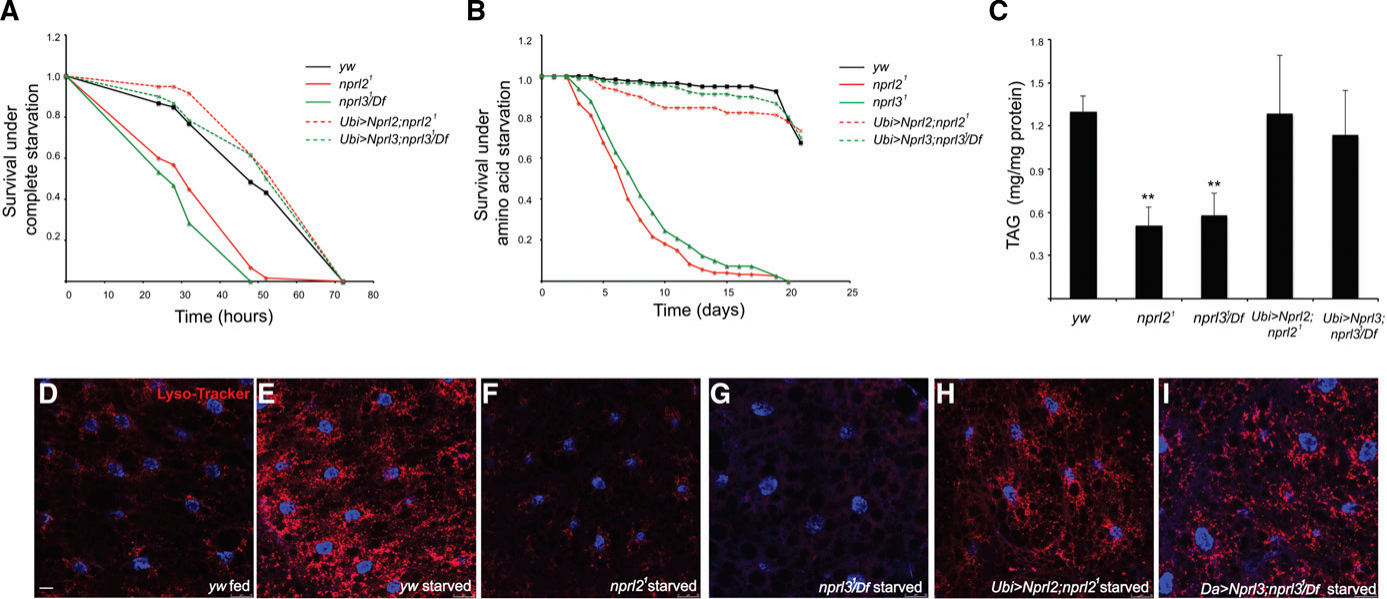
*nprl2* and *nprl3* mutants are sensitive to starvation. (A) Newly hatched male flies were cultured at 25° on standard food for 3 d, and then transferred to starvation media (0.8% agar in PBS) and counted at the indicated time points. The survival curves of *wild-type*, *nprl2^1^*, *nprl3^1^/Df*, *Ubi-GAL4/UAS-Nprl2*; *nprl2^1^*, and *Ubi-GAL4/UAS-Nprl3*; *nprl3^1^/Df* are shown. (B) Newly hatched male flies were cultured at 25° on standard food for 3 d, and then transferred to amino acid starvation media (20% sucrose, 0.8% agar in PBS) and counted each day. The survival curves of *wild-type*, *nprl2^1^*, *nprl3^1^/Df*, *Ubi-GAL4/UAS-Nprl2*; *nprl2^1^*, and *Ubi-GAL4/UAS-Nprl3*; *nprl3^1^/Df* are shown. (C) Newly hatched male flies were cultured at 25° on standard food for 3 d and then lysed. Total body TAG and protein were measured. Protein levels were used for normalization. The TAG to protein ratio is shown. Error bars indicate SD of three independent experiments. ** *P* < 0.01. (D–I) Third instar *wild-type*, *nprl2^1^*, *nprl3^1^/Df*, *Ubi-GAL4/UAS-Nprl2*; *nprl2^1^*, and *Da>-GAL4/UAS-Nprl3*; *nprl3^1^/Df* larvae were fed or starved for 4 hr. Subsequently, fat bodies were removed and stained with Hoechst and LysoTracker Red. PBS, phosphate-buffered saline; TAG, triglycerides.

The GATOR1 complex downregulates TORC1 activity in response to nutrient stress triggering the activation of catabolic metabolism and autophagy (Chang *et al.* 2009; Neklesa and Davis 2009; Bar-Peled *et al.* 2013; Panchaud *et al.* 2013a). Previous work has shown that the GATOR1 complex promotes a cell autonomous response to nutrient stress in the female germline of *Drosophila* (Wei and Lilly 2014). Specifically, in the absence of GATOR1, egg chambers undergo apoptosis when females are cultured under conditions of amino acid restriction. In order to determine if *nprl2* and *nprl3* are required to mediate a robust response to nutrient stress in *Drosophila* at the organismal level, we examined the survival rates of *nprl2* and *nprl3* mutants under conditions of complete starvation and amino acid starvation (20% sucrose). As shown in Figure 3, A and B, the *nprl2* and *nprl3* mutants have decreased survival rates under both nutrient-limiting culture conditions. Thus, Nprl2 and Nprl3 promote starvation tolerance at the organismal level. In *Drosophila*, triglycerides (TAG) stored in the fat body are the primary energy resource and are essential to starvation resistance (Arrese *et al.* 2010). Consistent with increased sensitivity to starvation, *nprl2* and *nprl3* mutant adults contain reduced amounts of stored TAG relative to wild-type animals (Figure 3C). Importantly, the decreased survival in response to nutrient stress and the TAG storage deficit of *nprl2* and *nprl3* mutants were rescued by the ubiquitous expression of the corresponding transgene (Figure 3, A–C). These results are in line with previous reports that treatment with the TORC1 inhibitor Rapamycin results in increased stress resistance and elevated TAG levels (Bjedov *et al.* 2010).

The autophagy pathway promotes the digestion of nonessential cellular components to provide basic nutrients for cell survival during times of nutrient scarcity (Rabinowitz and White 2010). An important trigger for the activation of autophagy is the down-regulation of TORC1 activity (Kamada *et al.* 2010). We wanted to test the idea that the high TORC1 activity observed in the *nprl2* and *nprl3* mutants prevents the activation of autophagy, thus contributing to the starvation sensitivity of the mutants. LysoTracker, which highlights acidic compartments and thus stains lysosomes and autolysosomes, has been commonly used for examining autophagy in the *Drosophila* fat body, an organ that stores energy and is sensitive to nutrient status (Klionsky *et al.* 2016). In order to test this model, we stained mutant and wild-type fat bodies from third instar larvae with LysoTracker. In wild-type fat body cells, amino acid starvation results in the accumulation of lysosomes/autolysosomes as indicated by the accumulation of LysoTracker-positive puncta (Scott *et al.* 2004; Mauvezin *et al.* 2014) (Figure 3, D and E). In contrast, fat bodies from *nprl2* and *nprl3* mutant larvae fail to accumulate large numbers of LysoTracker-positive puncta in response to amino acid starvation (Figure 3, F and G). The failure to activate autophagy in the *nprl2* and *nprl3* mutants is rescued by the ubiquitous expression of the corresponding transgene. Thus, our data are consistent with the model that *nprl2* and *nprl3* mutants fail to activate catabolic metabolism and autophagy in response to amino acid starvation because of the failure to downregulate TORC1 activity. Notably, a similar phenotype is observed in mutants of the potent TORC1 inhibitor *tuberous sclerosis complex* (TSC) (Scott *et al.* 2004). In *Drosophila*, autophagy is essential for the response to nutrient deprivation (Scott *et al.* 2004; Lenardo *et al.* 2009; Neufeld 2010). Thus, we predict that the increased susceptibility to starvation observed in *nprl2* and *nprl3* mutants may be due to both decreased TAG storage and the inability to downregulate TORC1 activity and activate the autophagy pathway during periods of nutrient scarcity.

### Nprl2 and Nprl3 function to promote Drosophila motility

**Figure 4:**
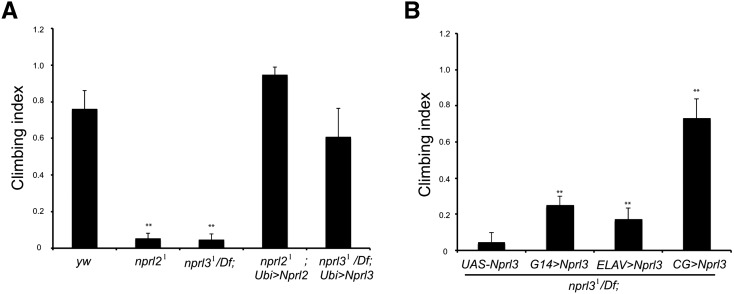
Nprl2 and Nprl3 are required in the fat body for *Drosophila* locomotion. (A) The climbing indices of the indicated genotypes are shown. Overexpression of Nprl2 and Nprl3 using the ubiquitous *Ubi-GAL4* driver suppressed the climbing defects of *nprl2^1^* and *nprl3^1^/Df* mutant flies. Error bars indicate SD of at least three independent experiments. ** *P* < 0.01. (B) The climbing indices of *nprl3^1^/Df* mutant flies that overexpress the Nprl3 protein using a neuronal driver ELAV-GAL4, a muscle driver G14-GAL4, or a fat body, hemocyte driver CG-GAL4 are shown. Error bars indicate SD of at least six independent experiments. ** *P* < 0.01.

During the course of our studies, we noted that *nprl2* and *nprl3* mutants had reduced motility. We quantified this motility defect using a standard geotaxis-climbing assay (Ganetzky and Flanagan 1978; Feany and Bender 2000). Consistent with our initial observations, *nprl2* and *nprl3* mutant adults had reduced climbing indices relative to wild-type controls (Figure 4A). The climbing deficits were rescued by the expression of the *nprl2* or *nprl3* transgene from a ubiquitous *Ubi-GAL4* driver in the respective mutant backgrounds (Figure 4A). There are at least two possible models for how the GATOR1 complex might affect *Drosophila* motility. First, GATOR1 may be required cell autonomously for the development or the maintenance of metabolic homeostasis in neurons and muscles. Notably, several *Drosophila* neurodegenerative disease models have locomotive defects that can be rescued through genetic inhibition of TORC1 in neurons and muscle (Tain *et al.* 2009; Hirth 2010; Liu and Lu 2010). Alternatively, GATOR1 may act nonautonomously to control the systemic metabolism and energy availability that are required for motility. The *Drosophila* fat body plays a critical role in maintaining metabolic homeostasis (Colombani *et al.* 2003; Arrese and Soulages 2010). In the presence of amino acids, TORC1 activity in the fat body promotes global growth and anabolic metabolism through a noncell autonomous insulin-dependent pathway (Colombani *et al.* 2003). To distinguish between these two possibilities, we compared the climbing index of *nprl3* mutant flies that expressed Nprl3 from a transgene in neurons and muscle using the neuronal and muscle drivers ELAV-GAL4 and G14-GAL4 (Aberle *et al.* 2002) to mutants that expressed Nprl3 in the fat body and hemocytes using the driver CG-GAL4 (Asha *et al.* 2003). Somewhat surprisingly, we found that while the expression of Nprl3 in neurons and muscle resulted in a small increase in the climbing index, the expression of Nprl3 in the fat body and hemocytes resulted in a near full rescue of the *nprl3^1^/Df* mobility deficits (Figure 4B). These data strongly suggest that the activity of the GATOR1 complex in the fat body is required to promote adult motility. Furthermore, we found that overexpression of Nprl3 in the fat body and hemocytes can rescue the lethality of *nprl3* mutants (Table 2). Taken together, our data indicate that the GATOR1 complex has both autonomous and nonautonomous functions in the regulation of growth and metabolism in *Drosophila*.

### Conclusions

In summary, we have defined the developmental and metabolic role of the newly identified TORC1 inhibitor GATOR1 in the genetically tractable animal model *D. melanogaster*. We have shown that the GATOR1 complex regulates multiple aspects of cell growth and metabolism, influencing processes as diverse as fat storage, growth, autophagy, and motility. Additionally, we found that the GATOR1 complex has both cell autonomous and nonautonomous functions. Taken together, our findings define *Drosophila* as an excellent model for the genetic dissection of the GATOR1 complex and its role in preventing the developmental defects and pathologies associated with deregulated TORC1 activity.

## Supplementary Material

Supplemental Material
