# Supplementary material for: The GATOR1 Complex Regulates Metabolic Homeostasis and the Response to Nutrient Stress in Drosophila melanogaster
Source: G3 (Bethesda). 2016 Sep 26;6(12):3859–67. doi: 10.1534/g3.116.035337 (PMC5144957; doi:10.1534/g3.116.035337)
Supplement: Supplemental Material [file supp_6_12_3859__index.html]

The GATOR1 Complex Regulates Metabolic Homeostasis and the Response to Nutrient Stress in Drosophila melanogaster — Supplemental Material 

# The GATOR1 Complex Regulates Metabolic Homeostasis and the Response to Nutrient Stress in *Drosophila melanogaster*

## Supplemental Material for Wei, *et al*, 2016

**Files in this Data Supplement:**

- Table S1 - Primers used for generating and screening GATOR1 mutants. (.pdf, 64 KB)
- Figure S1 - The GATOR1 mutants are lethal at the pupae/pharate adult stage. (.jpg, 430 KB)
