# Supplementary material for: The GATOR1 Complex Regulates Metabolic Homeostasis and the Response to Nutrient Stress in Drosophila melanogaster
Source: G3 (Bethesda). 2016 Sep 26;6(12):3859–67. doi: 10.1534/g3.116.035337 (PMC5144957; doi:10.1534/g3.116.035337)
Supplement: Supplemental Material [file supp_g3.116.035337_TableS1.pdf]

Table S1. Primers used for generating and screening GATOR1 mutants.

Primers	Sequence
Nprl2 5’ side guide RNA Forward	CTTCGGGTGCCCATGGCAGCACGG
Nprl2 5’ side guide RNA Reverse	AAACCCGTGCTGCCATGGGCACCC
Nprl2 3’ side guide RNA Forward	CTTCGGGCGGCAGAAGATGTACAC
Nprl2 3’ side guide RNA Reverse	AAACGTGTACATCTTCTGCCGCCC
Iml1 5’ side guide RNA Forward	CTTCGGACTTGGTGATGAATCTAA
Iml1 5’ side guide RNA Reverse	AAACTTAGATTCATCACCAAGTCC
Iml1 3’ side guide RNA Forward	CTTCGATAAACAACTCCGCGGACG
Iml1 3’ side guide RNA Reverse	AAACCGTCCGCGGAGTTGTTTATC
Nprl2 deletion detected primer Forward	CCGCTTATCCAGTTGGCAGA
Nprl2 deletion detected primer Reverse	TGTGGATGCGGTGATACTGG
Nprl3 deletion detected primer Forward	CCAGAGTTTCGTAGGCAGGA
Nprl3 deletion detected primer Reverse	CAAGGGGGATCGGTTACTGT
Iml1 deletion detected primer Forward	CAGTCAATAGCAACGCACC
Iml1 deletion detected primer Reverse	CTATTTCATCTTATTCTCCTC
FRT detection Primer Forward	CGACCACCAAGCGAAACATC
FRT detection Primer Reverse	ATCTCGTGATGGCAGGTTGG
