# Supplementary figures and images for: The GATOR1 Complex Regulates Metabolic Homeostasis and the Response to Nutrient Stress in Drosophila melanogaster
Source: G3 (Bethesda). 2016 Sep 26;6(12):3859–67. doi: 10.1534/g3.116.035337 (PMC5144957; doi:10.1534/g3.116.035337)

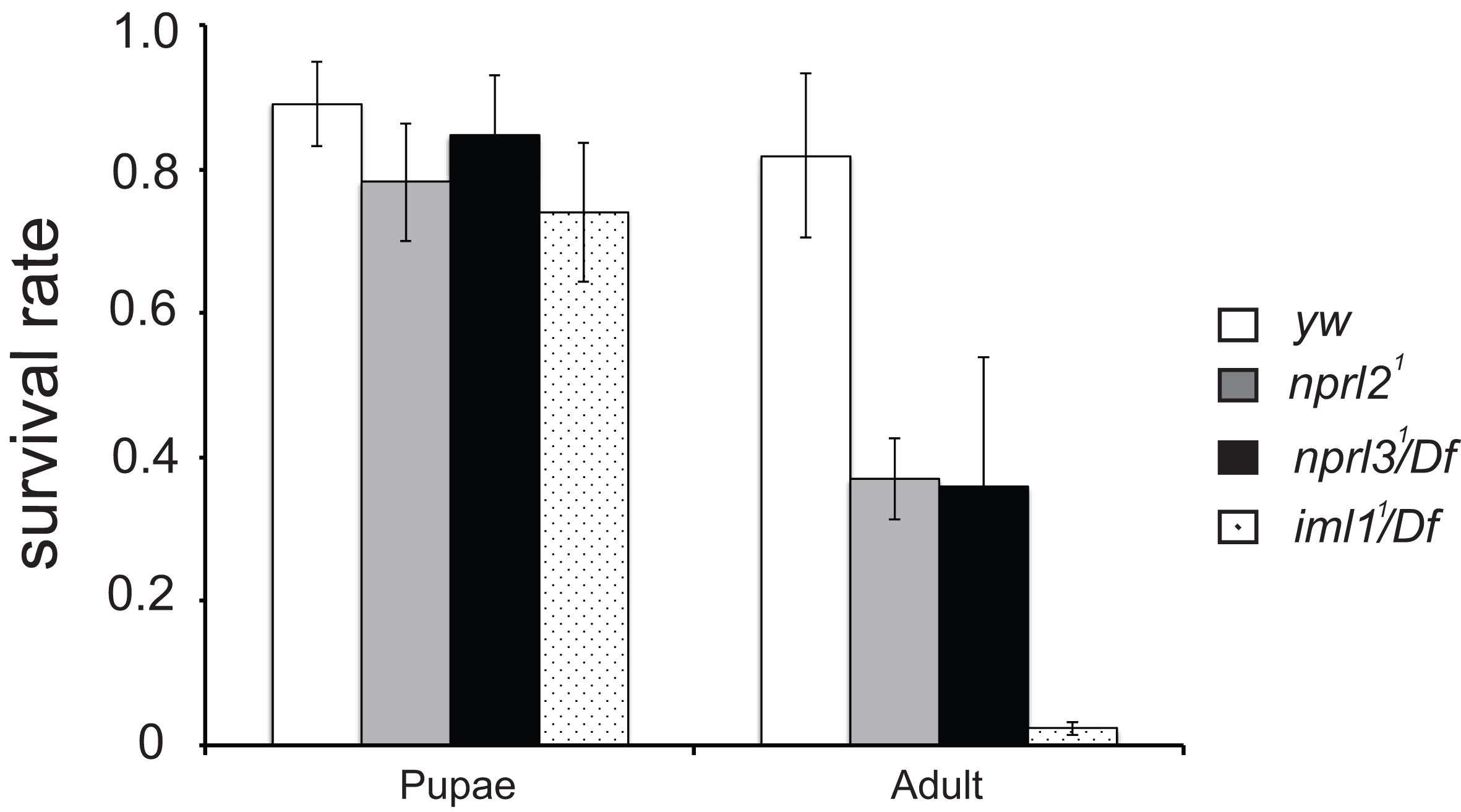

Supplement: Supplemental Material [file supp_g3.116.035337_FigureS1.jpg]
